# Novel antibiofilm chemotherapies target nitrogen from glutamate and glutamine

**DOI:** 10.1038/s41598-018-25401-z

**Published:** 2018-05-08

**Authors:** Tal Hassanov, Iris Karunker, Nitai Steinberg, Ayelet Erez, Ilana Kolodkin-Gal

**Affiliations:** 1 Department of Molecular Genetics, Weizmann Institute of Science, Rehovot, Israel; 2 Department of Biological Regulation, Weizmann Institute of Science, Rehovot, Israel

## Abstract

Bacteria in nature often reside in differentiated communities termed biofilms, which are an active interphase between uni-cellular and multicellular life states for bacteria. Here we demonstrate that the development of *B*. *subtilis* biofilms is dependent on the use of glutamine or glutamate as a nitrogen source. We show a differential metabolic requirement within the biofilm; while glutamine is necessary for the dividing cells at the edges, the inner cell mass utilizes lactic acid. Our results indicate that biofilm cells preserve a short-term memory of glutamate metabolism. Finally, we establish that drugs that target glutamine and glutamate utilization restrict biofilm development. Overall, our work reveals a spatial regulation of nitrogen and carbon metabolism within the biofilm, which contributes to the fitness of bacterial complex communities. This acquired metabolic division of labor within biofilm can serve as a target for novel anti-biofilm chemotherapies

## Introduction

Despite the widely held view of bacteria as unicellular organisms that struggle for individual survival, in nature, bacteria establish complex communities, referred to as biofilms. Biofilm are differentiated communities, where the inhabitant cells are held together by an organic extracellular matrix^1–3^ and biogenic minerals^4–6^. In a biofilm, cells use a variety of mechanisms to coordinate activity within the community, as well as across species^7,8^. In many instances, biofilms provide beneficial effects to other organisms, e.g., biocontrol agents form biofilms on the surface of plant roots to prevent the growth of bacterial and fungal pathogens^9–12^. In other situations, biofilms can have deleterious effects; in a clinical context, biofilms are inherently resistant to antimicrobial agents, and are at the core of many persistent and chronic bacterial infections^13^. Thus, gaining insights into microbial sociology and biofilm formation, will potentially provide significant clinical^13,14^, technological^15–17^, agricultural^10,18^ and ecological^19–21^ advancements.

The Gram-positive bacterium *Bacillus subtilis* is a robust biofilm former, and can form a structured biofilm colony comprised of cells encapsulated by a thick extracellular matrix on a solid–air interface^7,22^. The resultant colony morphology is considered a direct readout for differentiation and extracellular matrix production^22–24^.

So far we and others uncovered several cues that have been associated with biofilm colonies maturation and assembly, including depravation of oxygen and trace elements^25–29^, small molecule sensing^30–32^, calcium^6,33^, and physical signals^34–37^. And yet, there are other developmental cues awaiting to be discovered.

Although derived from a single genetically identical clone, bacteria within mono-species biofilms are heterogeneous in terms of metabolism, gene expression and physiology, creating diverse biological niches within the biofilm^7,38^. This heterogeneity facilitates response to changing environmental conditions, allowing survival and growth of the biofilm community^39,40^. With time, multiple division events take place, the biofilm thickens, and the founder population generates the inner cell mass, while the dividing cells tend to be localized to the edges^41,42^. The differential metabolic activities of the cells in the inner mass, periphery and transition areas result in concentration gradients of nutrients. As a result, cells that are growing in the different areas of the biofilm become very distinct from each other. As biofilm development proceeds, its survival requires the establishment of complex spatial associations between the periphery and inner cells^41^. Several recent findings linked the development of bacterial multicellular communities with the metabolism of glutamine and glutamate in the Gram-positive bacterium *B*. *subtilis*^26,37,43,44^. Glutamine is a nonessential and abundant amino acid which can be converted to ammonia and glutamate, providing an important source of carbon and nitrogen for the synthesis of nucleic acids, amino sugars, and proteins. Glutamine can be acquired from the growth media or generated by glutamine synthase from glutamate and ammonia. Recently it was shown that in the biofilm, glutamate is up taken from the medium by the peripheral cells while only the inner cells have the ability to synthesize ammonia^43^. This co-dependence between the peripheral and central cells for glutamine synthesis gives rise to metabolic commensalism; the peripheral cells growth is restricted so that they cannot consume all the glutamate and starve the inner cells, because they depend on the inner cells to synthesize ammonia for glutamine synthesis. In parallel, glutamate dehydrogenase paralogous GudB and RocG which catalyze glutamate, contribute to the fitness of biofilm cells and interference with their transcriptional regulation has deleterious effects^44^. While both glutamine and glutamate were shown to be important to biofilm fitness, their specific contribution to biofilm development remains to be determined. Specifically, it is still unknown whether they provide mainly carbon, nitrogen, or both. Here we systematically explore the metabolic contributions of glutamate and glutamine to biofilm development for translational and therapeutic applications.

## Materials and Methods

### Strains

Experiments were performed with *Bacillus subtilis* NCIB 3610^22^ and its indicated derivatives, *Enterococcus faecalis* 29212^45^, and *Pseudomonas aeruginosa PA01*^46^.

### Construction of strains

Laboratory strains *B*. *subtilis* PY79 and *E*. *coli* DH5α were used for cloning purposes. Transformation of *B*. *subtilis* PY79 with linearized plasmid or PCR products, was performed as previously described^47^.

For P_*glnRA*_ fusions to GFP and LacZ we used the following primers: a forward primer GATCGAATTCATTTTTAAAATTTCTCTGGATTG, and a reverse primer: TGCGAAGCTTGGTAAAATTCCTCCTCTTAA. For P_*ldh*_ fusion to GFP we used the following primers: a forward primer GTGTGAGAATTCAGTTTTGTTAAAAGAGATCCAGCG, and a reverse primer: TGC GTCAGTAAGCTTCATTAATCATCCTTGCAGGGT.

The plasmid pYC121^48^ which contains a functional GFP gene and a chloramphenicol resistance gene was used as a template for the construction of GFP reporter strains. The plasmid pDG1728^49^ which contains a functional β-galactosidase gene and a spectinomycin resistance gene was used as a template for the construction of LacZ reporter strains. PCR fragments were amplified from NCIB 3610 chromosomal DNA, using primers with the suitable restriction sites for ligation into the plasmid. The ligated plasmids were then transformed into *E*. *coli* DH5α and ampicillin resistant colonies were selected and confirmed by sequencing. The reporters were then integrated into the neutral *amyE* locus of strain NCIB 3610 by transformation, as described above, and selected for antibiotic resistance. Strains carrying *tnrA* deletion and the P_*ldh*_*-lacZ* were generated by us previously from the parental strain NCIB 3610^26,27^.

### Growth media

The strains were routinely manipulated in biofilm medium, containing MS salts and 125 µM FeCl_3_^26^, either with glycerol and glutamate (MSgg^26^) or as indicated in each figure legend. When grown anaerobically, a nitrate source (KNO_3_) was added to the medium as previously described^27^. The solid medium contained 1.5% bacto agar (Difco).

### Biofilm development assay

A single *B*. *subtilis* colony, isolated on solid LB plates, was used to inoculate 3 ml LB broth starter culture and grown to mid-logarithmic phase at 37 °C. Then, a 2 μL drop of the culture was spotted on MSgg-nitrate solid medium. Plates were incubated at room temperature. Images and GFP signal intensity were obtained with a Stereo Discovery V20″ microscope with Objective Plan Apo S 0.5xFWD 134 mm or Apo S 1.0x FWD 60 mm (Zeiss) attached to an Axiocam camera. Data were analyzed using Axiovision suite software (Zeiss).

### Biofilm imaging

To grow biofilms, 1.5 µl of starter culture was inoculated on plates of solid MSgg medium that were dried in a biological hood for 45 min prior to inoculation. Plates were grown at 30 °C. Photos of colonies were acquired with a NikonD800 camera or a stereomicroscope (Zeiss) and images were optimized for contrast and brightness using Adobe Photoshop.

### β-galactosidase assay

Biofilms were grown on solid MSgg medium as indicated. The colonies were collected, resuspended in 1 mL PBS, and sonicated to remove the extracellular matrix (BRANSON digital sonifier, Model 250, Microtip, amplitude 20%, pulse 3x 5 sec). OD_600_ had been measured (Ultrospec 2100, Amersham Biosciences. Cells (10^8^–10^9^) were taken for the assay. Cells were spun down, and pellets were resuspended in 1 mL of Z buffer (40 mM NaH_2_PO_4_, 60 mM Na_2_HPO_4_, 1 mM MgSO_4_, 10 mM KCl, 38 mM β-mercaptoethanol) supplemented with 200 μg mL^−1^ freshly made lysozyme. The samples were incubated for 15 min at 30 °C. Reactions were started by adding 200 μL of 4 mg mL^−1^ ONPG (2-nitrophenyl β-D-galactopyranoside) and stopped by adding 500 μL of 1 M Na_2_CO_3_. The soluble fractions were transferred to cuvettes (VWR), and OD_420_ values of the samples were recorded using a Pharmacia Ultraspectrometer 2000. The β-galactosidase-specific activity was calculated according to the equation [(OD_420_/(time × OD_600_)] × dilution factor × 1000. Time represents the time follwing the addition of ONPG. Assays were conducted in triplicates. For anaerobic growth and anoxic growth, biofilms were grown in anaerobic chamber or by the candle jar method^50^ in the presence of (KNO_3_)^27,51^.

### Crystal violet essay

To grow biofilms, 1.5 µl of starter culture was inoculated into TSB (Difco) (for *P*. *aeruginosa*) or TSB-Glucose media (for *E*. *faecalis)* in 96 well polystyrene plates either with or without AOA and DON. Plates were treated later as discussed in the corresponding figure legend. Crystal violet assay (Sigma) was performed as described previously^52^.

### Gas chromatography/mass spectrometry (GC/MS)

Biofilm cells were collected from three independent colonies, from the periphery and center, as previously described^26^. Cells were mildly sonicated to remove the extracellular matrix portion, and the optical density (OD_600_) of the collected fraction was measured. Cells were then washed with ice cold saline and lysed with 50% methanol in water followed by three freeze thaw cycles in liquid nitrogen. The insoluble material was pelleted in a cooled centrifuge (4 °C) and the supernatant was collected for subsequent GC-MS analysis. Samples were dried under air flow at 42 °C using Techne Dry-Block Heater with sample concentrator (Bibby Scientific Limited, UK). Dried samples were treated with 40 μl methoxyamine hydrochloride solution (20 mg/ml in pyridine) at 37 °C for 90 min while shaking, followed by incubation with 70 μl N, O-Bis (trimethylsilyl) trifluoroacetamide (Sigma) at 37 °C for additional 30 min. The samples were centrifuged and allowed to stand at room temperature for 2 hours before injection. The results were normalized to ribitol as an internal standard and to the OD_600_ absorption of each sample. GC/MS analysis was performed using a gas chromatograph (7820AN, Agilent Technologies, USA) interfaced with a mass spectrometer (5975 Agilent Technologies, USA). A HP-5ms capillary column 30 m × 250 µm × 0.25 µm (19091S-433, Agilent Technologies, USA) was used. Helium carrier gas was maintained at a constant flow rate of 1.0 mL min^−1^. The GC column temperature was programmed from 70 to 150 °C via a ramp of 4 °C min^−1^, 150–215 °C via a ramp of 9 °C min^−1^, 215–310 °C via a ramp of 25 °C min^−1^ and maintained at 310 °C for additional 5 min. The MS was done by electron impact ionization and operated in full scan mode from *m*/*z*, 30–500. The inlet and MS transfer line temperatures were maintained at 250 °C and 310 °C, respectively. The ion source temperature was 280 °C. Sample injection (1–3 μL) was in splitless mode.

### Planktonic growth analysis

Cells were diluted 1:100 in 150 µl liquid MSgg medium of each well of a 96-well plate (Thermo Scientific). Cells were grown with agitation at 30 °C for 12 hours in a microplate reader (Synergy 2, BioTek), and the optical density at 600 nm (OD_600_) was measured every 30 min. Cells were grown in a relevant growth medium as indicated in the corresponding figure legend.

### Cell density analysis of biofilms

To determine culture density of cells grown in biofilm colonies, cells were harvested from a biofilm colony (described above), and thoroughly vortexed. Untreated wild type cells were mildly sonicated (BRANSON digital sonifier, Model 250, Microtip, amplitude 20%, pulse 3x 5 sec). Optical density OD_600_ was measured with a spectrophotometer (Ultrospec 2100, Amersham Biosciences).

### Biofilm cells regrowth analysis

To evaluate regrowth of different subpopulations we harvested the biofilm cells from the center and the edges of a single colony into 500 µL of PBS solution (see Fig. S1) and performed mild sonication in BRANSON digital sonifier, Model 250, Microtip, amplitude 20%, pulse 3x 5 sec),as described previously^30,53–55^, and diluted to equivalent cell numbers in PBS. Experiments were performed with at-least three technical repeats (three individual colonies). To avoid bias we started all the experiments with equivalent number of cells from either the center or the edge (OD = 0.005) in the indicated growth media.

## Results

**Figure 1:**
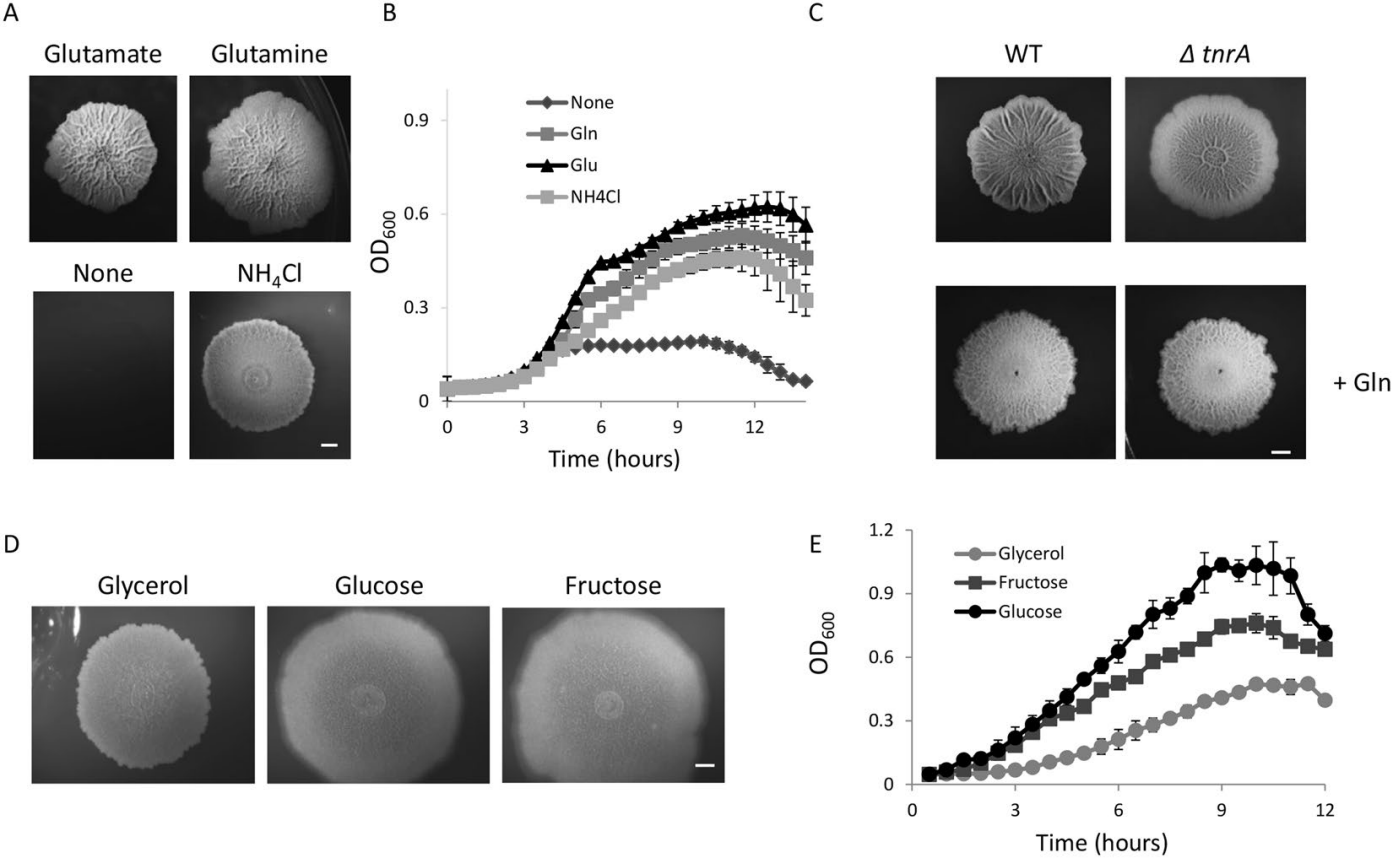
Glutamate and glutamine is an essential nitrogen source for biofilm development. (**A**) Biofilms were grown on solid medium containing MS salts and glycerol as a carbon source as described in the materials and methods, to which different nitrogen sources were added; glutamate, glutamine (upper panel), ammonium chloride or no nitrogen source (bottom panel). The nitrogen sources were applied in an equimolar ratio to the original glutamate concentration in MSgg medium. Biofilms were grown for three days at 30 °C. Images of colonies and pellicles were acquired with a stereomicroscope (Zeiss) and were optimized for contrast and brightness, with adjustments kept consistent between control and treatments. Scale bar corresponds to 2 mm. (**B**) Planktonic growth of wild type cells grown at 30 °C with shaking in liquid biofilm medium. Planktonic growth in medium supplemented with different nitrogen sources was monitored by measuring OD_600_ in a microplate reader. Results reflect averages of seven wells within one experiment. Errors bars represent the corresponding standard deviations. A representative of six independent experiments is shown. (**C**) TnrA, a transcription factor that coordinates glutamine and glutamate synthesis has a role in biofilm development. Parental wild-type strain and its *tnrA* mutant derivative were grown on either MSgg (top), or MSgg supplemented with 1% glutamine (bottom) for three days. Scale bar corresponds to 2 mm. (**D**) The effect of altering carbon sources on biofilm formation Biofilms were grown on solid medium containing MS salts, ammonium chloride and the indicated carbon sources (0.5%) as described in the materials and methods. Biofilms were grown for three days at 30 °C. Images of colonies were acquired with a stereomicroscope (Zeiss) and were optimized for contrast and brightness, with adjustments kept consistent between control and treatments. Scale bar corresponds to 2 mm. (**E**) Planktonic growth of wild type cells grown at 30 °C with shaking in liquid. Planktonic growth in the biofilm media supplemented with different nitrogen sources was monitored by measuring OD_600_ in a microplate reader. Results reflect averages of five wells within one experiment together. Errors bars represent the corresponding standard deviations. A representative of three independent experiments is shown. All curves differed from each other with a p-value < 0.005.

Glutamine and glutamate are important nitrogen and carbon sources for most multicellular organisms^56^. Thus, we first evaluated the necessity of both glutamine and glutamate for bacterial biofilm development by measuring biofilm maturation, biomass and planktonic growth, following supplementation of different nitrogen sources to the biofilm defined media. In the traditional biofilm defined medium which contains glutamate or glutamine as the primary nitrogen source, we found that *B*. *subtilis* wild type strains form an almost perfect circular biofilm shape, characterized by thick branching wrinkles that surround a defined circular center with smaller, more delicate, and denser creases (Fig. 1A upper panel). However, medium with neither of these two metabolites led to no biofilm development, while nitrogen salt supplementation, resulted in less mature biofilm formation, lacking pronounced wrinkles and ridges (Fig. 1A lower panels). Interestingly, this requirement was specific for biofilm development, as all three nitrogen sources were sufficient to sustain planktonic growth (Fig. 1B).

In the biofilm, glutamine levels are regulated by the activity of TnrA^57–59^. As expected from our findings, mutating *tnrA* led to a developmental defect in biofilms of *B*. *subtilis* without affecting planktonic growth (Figs 1C and S2). To test whether the induced defect in TnrA altered the ability of the biofilm to utilize glutamine, we grew the wild type its *tnrA* mutant derivative, with medium containing high levels of glutamine. In the presence of high concentration of glutamine, the *tnrA* mutant made a rouges colony enriched with small dense wrinkles, comparable to the parental strain grown under the same conditions (Fig. 1C). This clear inability of *tnrA* to develop a mature colony in the absence of glutamine suggests that cells depend on TnrA for glutamine synthesis, further correlating glutamine availability with transcription regulation and highlighting its role biofilm development.

To test whether nitrogen contributes to biofilm formation more than carbon, we tested whether a transition between non-favorable carbon source (glycerol) and favorable carbon sources (fructose and glucose)^60^ in minimal media has a similar effect on biofilm development. In contrast to the effects of different nitrogen sources on biofilm development shown in Fig. 1A,B, we found that alterations of the carbon sources had more subtle effects on colony morphology, and yet dramatically affected planktonic growth (Fig. 1D,E). These results together suggest that while the biofilm development depends on the specific source of nitrogen, planktonic growth depends more on the availability and source of carbon.

To distinguish between the contribution of glutamine and glutamate to biofilm development, we used two inhibitors for glutamine and glutamate synthesis 6-Diazo-5-oxo-L-norleucine (DON)^61^, a glutamine analogue, and aminooxyacetic acid (AOA), an inhibitor of glutamate oxaloacetate transaminase and aspartate aminotransferase, which restricts glutamate and its downstream metabolites’ levels^62,63^. These drugs are used for cancer therapy as most cancer cells depend on glutamine for growth^64^, but were also suggested to affect bacterial planktonic growth, and nitrogen metabolism^61,62^. Based on our findings described here for the similar biofilm dependence on glutamine and glutamate, we hypothesized that these drugs would inhibit biofilm development.

**Figure 2:**
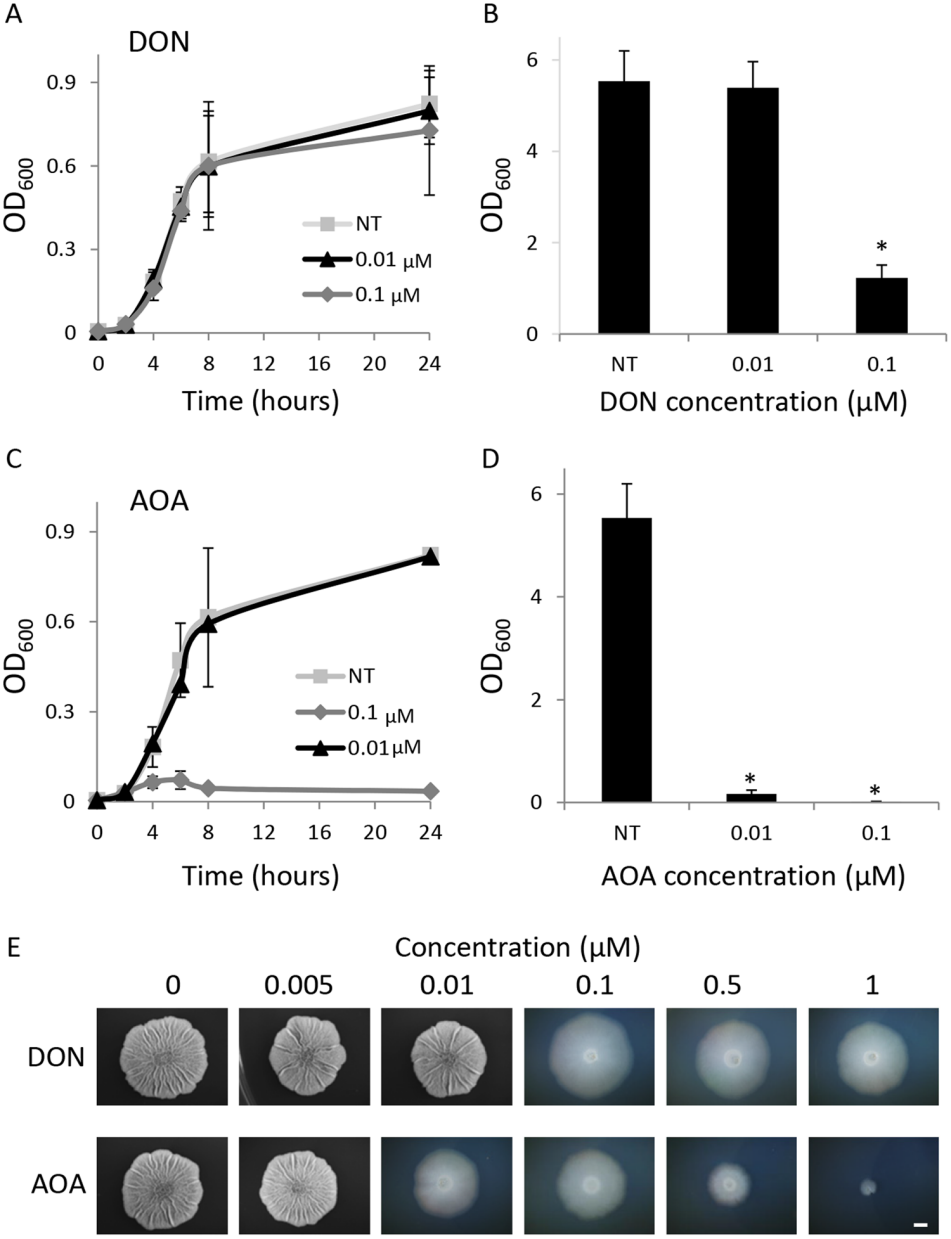
Targeting glutamate and glutamine synthesis inhibits biofilm formation. (**A**) Planktonic growth of bacteria grown in MSgg medium with or without different concentrations of DON (µm) was monitored by measuring OD_600_ in a microplate reader. Results shown are averages of seven wells within one experiment and their corresponding standard deviations. A representative of six independent experiments is shown. (**B**) Biofilms were grown on solid MSgg medium in the presence of the indicated concentrations of DON (µM) for three days at 30 °C. After three days, cells were harvested, homogenized (see Materials and Methods) and cell density was determined by measuring OD_600_. Averages triplicates of one experiment are shown. Errors bars represent the corresponding standard deviations. This experiment is a representative of three independent experiments. (**C**) Planktonic growth of bacteria grown in MSgg medium with or without different concentrations of AOA (µM) was monitored by measuring OD_600_ in a microplate reader. Results shown are averages of seven wells within one experiment. Errors bars represent the corresponding standard deviations. A representative of six independent experiments is shown. (**D**) Biofilms were grown on solid MSgg medium, in the presence of the indicated concentrations of AOA (µM) for three days at 30 °C. After 3 days, cells were harvested, homogenized (see Materials and Methods) and cell density was determined by measuring OD_600_. Averages of triplicates of one experiment are shown. Errors bars represent the corresponding standard deviations. The experiment is a representative of three independent experiments. (**E**) Biofilms were grown on solid MSgg medium for three days at 30 °C in absence or presence of the indicated concentrations of DON and AOA (µM). Images of biofilm colonies were acquired with a stereomicroscope (Zeiss). Scale bar corresponds to 2 mm.

We measured the effects of these inhibitors on planktonic growth and on the biofilm biomass as a quantifiable readout for biofilm formation^65,66^. We found that the glutamine competitor DON at 100 nM concentration had little or no effect on planktonic growth and yet dramatically decreased the biomass of biofilm colonies (Fig. 2A,B). At the same dosage, AOA, acting downstream to glutamine and restricting glutamate levels, was extremely toxic to bacterial planktonic cultures (Fig. 2C), and affected the biofilm’s biomass growth more severely than DON (Fig. 2C,D). At the lower concentration (10 nM), both DON and AOA had no detectable effect on planktonic growth while AOA diminished biofilm biomass growth. These results support the dependence of the biofilm growth on glutamine and glutamate, as compared to planktonic growth. Moreover, biofilms that formed in the presence of sub-inhibitory concentrations of DON and AOA, were compromised in their formation of complex morphology, demonstrating a higher sensitivity to AOA (Fig. 2E). Excess glutamine reduced the sensitivity to DON (Table S1), and excess levels of both glutamine and glutamate had deleterious effects on cells growth but reduced the sensitivity to AOA (data not shown). Surprisingly, both DON and AOA primarily inhibited the development of the peripheral areas of the biofilm (Fig. 2E).

**Figure 3:**
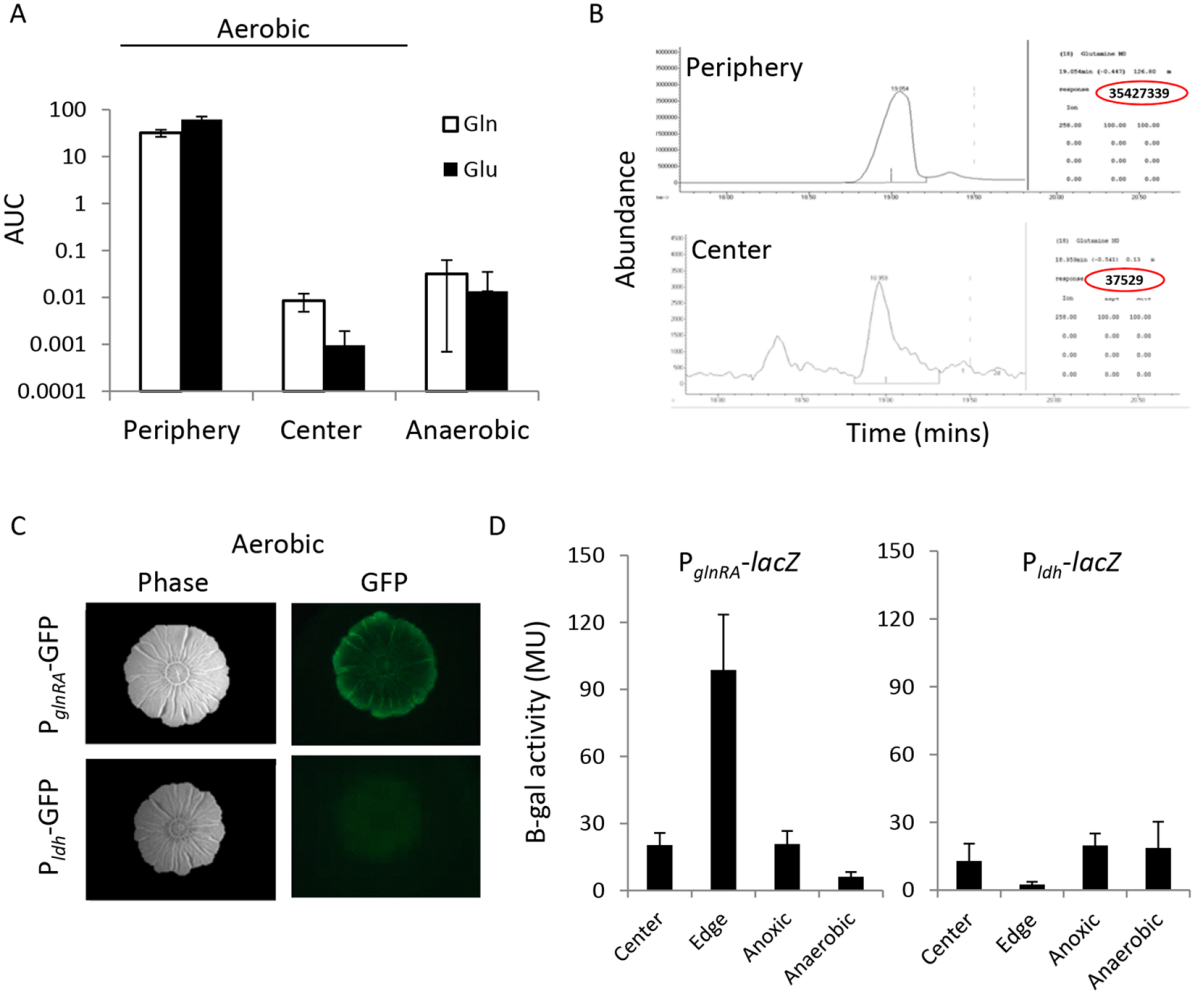
Spatial distribution of glutamate utilization within biofilms. (**A**) GC-MS analysis results for glutamine and glutamate levels in biofilm cells collected from different regions of the biofilms. The results are presented as averages and standard deviations of a representative experiment performed in quadruplicates, out of three independent biological repeats. The periphery samples for both metabolites differed from the anaerobic and center samples by a p–value < 0.0001. Anaerobic and center samples were not significantly different from each other (p-value > 0.5). (**B**) Representative measurements of MS ion intensities for glutamine in the periphery and in the center of *B*. *subtilis* biofilm colonies showing three order of magnitude higher levels of glutamine in the periphery (red circles point to the relevant specific values). (**C**) Biofilm strains carrying P_*glnR*_*-gfp* (the upstream promoter of the operon encoding glutamine synthase) or P_*ldh*_*-gfp* constructs (the promoter of lactate dehydrogenase) following 72 hours of growth on standard biofilm-inducing medium with nitrate under aerobic conditions. Images were taken with a stereo microscope using white light (left) or fluorescence (right). Shown is a representative experiment performed in duplicates, out of at least three independent repeats. (**D**) Expression of P_*glnRA*_-*lacZ* and P_*ldh*_-*lacZ*. Cells were grown on MSgg as described in Materials and Methods for 48 h. After 48 h, colonies carrying either P_*glnRA*_-*lacZ* or P_*ldh*_-*lacZ* were harvested, and LacZ activity was measured. The results are the average for three independent experiments performed with triplicates. Errors bars represent the corresponding standard deviations. For both reporters, the edge and the center were significantly different (P-value < 0.005).

Following the notion that the biofilm periphery and center cells differ in their requirement for glutamine and glutamate^43^, we quantified glutamine and glutamate levels in different layers of the biofilm, using mass spectrometry. We found significantly higher levels of both metabolites in the periphery than in the center (Fig. 3A,B). After differentiation, the peripheral regions in the biofilm were shown to rely on the TCA cycle, while the central regions were shown to rely on lactate utilization typical to anoxic growth^27,41,67,68^. Thus we hypothesized that the lower levels of glutamine and glutamate we find at the center of the biofilm, may correlate with increased levels of lactate. Indeed there was no difference in the levels of glutamine and glutamate between the central layer in aerobic conditions and a biofilm colony grown under anaerobic conditions (Fig. 3A,B). Importantly, under anaerobic conditions, morphogenesis is inhibited^27^ (Fig. S3). Consistent with the hypothesis that differential metabolism is mediated by morphogenesis, the concentrations of both lactate and glutamine were comparable in the center and edges during anaerobic conditions (an edge/center ratio of 0.6 folds for lactate, 1.4 folds for glutamate).

Under aerobic conditions, both lactate and glutamate, can serve as a carbon source, but only glutamate can also serve as a nitrogen source^69,70^. To directly evaluate whether the main dependence of the central biofilm is for carbon or nitrogen, we first assessed the correlation between the expression of glutamine synthase (*glnR)* and that of lactate dehydrogenase (*ldh)*. For this, we deduced *glnA* expression levels from the expression levels of two independent reporter genes [β-galactosidase and GFP] driven by the operon promoter (located upstream to the adjacent gene *glnR*^57^), as was done previously for other genes^44,55^. We found that *glnR* expression is higher in the periphery of the colony, while *ldh* expression was higher in the center (Fig. 3C,D). Furthermore, supplementing glutamate to the medium enabled full biofilm development, while supplementation of lactate alone led to the formation of colonies that lacked complex morphologies (Fig. S4). Importantly, when grown in anoxic and anaerobic conditions, in which glycolysis becomes prominent, *glnR* expression decreased, while *ldh* expression increased (Fig. 3D).

These results support the previously described phenotype for the biofilm center of mutants for lactate metabolism^67^ and suggest that while both glutamate and lactate support biofilm growth, only glutamate, enables the formation of a wrinkled colonies. Thus, nitrogen is likely more essential than carbon for the development of the complex 3D morphology of the biofilm colonies. The spatial differences in activation of different metabolic reactions as reflected by differential gene expression between the periphery and central cells of the biofilm, might suggest that lactic acid fermentation by the inner cell mass of bacterial biofilms specifically in anaerobic conditions, plays a compensatory metabolic role to their utilization of carbon from glutamine.

**Figure 4:**
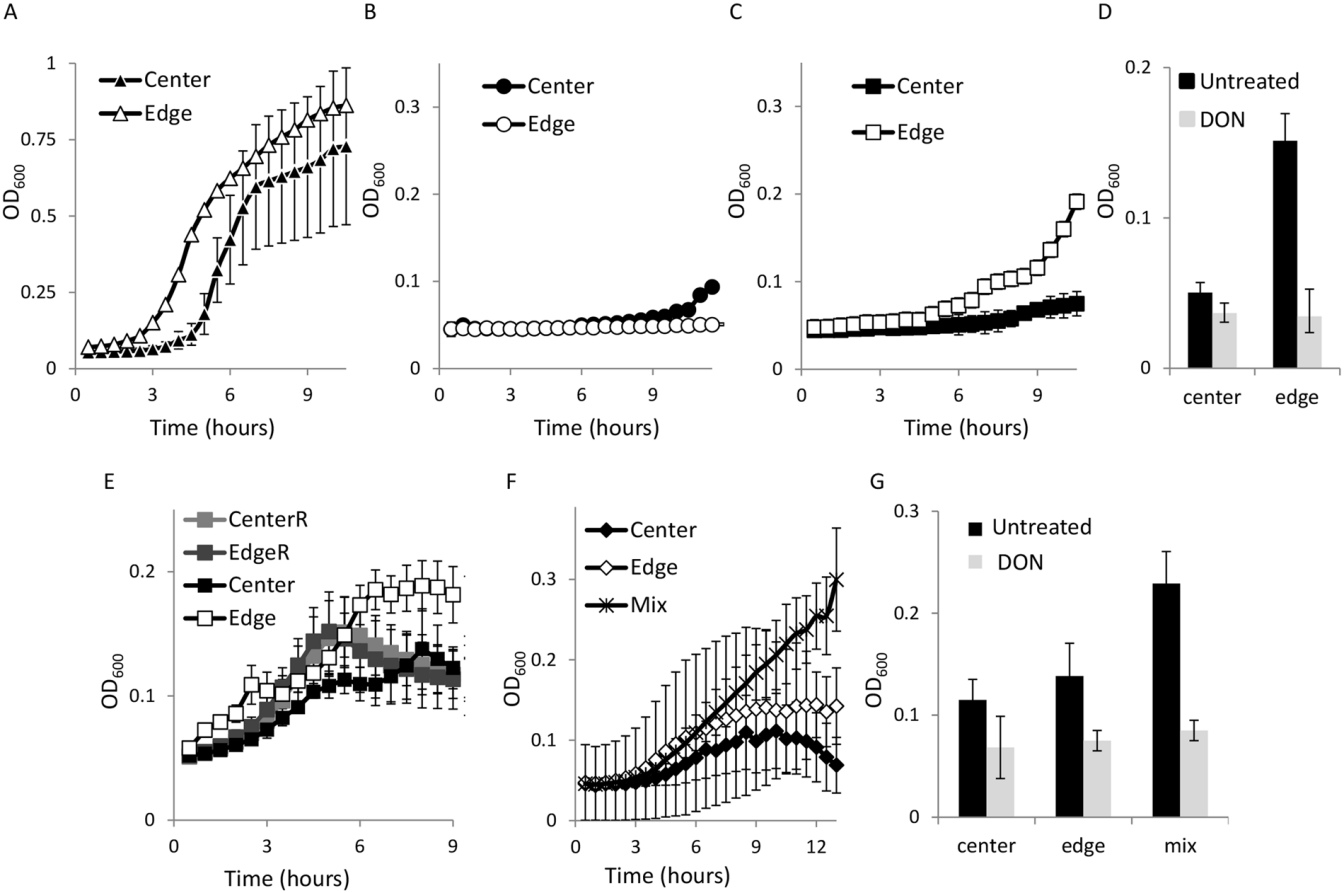
Spatial adaptation of biofilm cells to glutamate utilization. (**A**–**C**) Cells were harvested from developed biofilms indicated areas into PBS buffer, and gently sonicated. The cell counts were normalized by spectrophotometer_._ Harvested cells were inoculated in different media to assess planktonic growth by measuring OD_600_ in a microplate reader. (**A**) Tryptic Soy Broth (TSB) rich medium (Difco). (**B**) MS salts applied with glutamate where it serves as the sole carbon and primary nitrogen source. (**C**) MS medium with ammonium chloride as a nitrogen source and sodium lactate as a carbon source (0.5%). When grown on glutamate or sodium-lactate, the periphery (edge) samples differed from the center interior samples by P–values < 0.0001 and < 0.001 respectively by a two tailed t-test. Results are an average of a single experiment performed with five independent technical repeats. Errors bars represent the corresponding standard deviations. The experiment is a representative experiment of three independent repeats. When grown in non-limiting medium for glutamate, the periphery (edge) samples and center samples were not significantly different from each other (p-Value > 0.1). (**D**–**F**) Biofilm cells were harvested from the periphery [edge] or interior [center] into MS salts as follows: (**D**) Cells were grown with glutamate as the nitrogen source and either with or without DON (0.1 µM) for 10 hours. The culture grown either with or without DON differed from each other by a p-value < 0.005. The results are an average of two independent experiments performed with 10 technical repeats. The results are the average for three independent experiments performed with triplicates. Errors bars represent the corresponding standard deviations. (**E**) MS salts applied with glutamate where it serves as the sole carbon and primary nitrogen source. Cells were either inoculated directly from different zones of the biofilm as in B, or recovered for 4 hours in LB and diluted 1:100 in the glutamate-based medium (labeled as EdgeR and CenterR for their independent sources). The results are an average of three technical triplicates per three independent colonies. Errors bars represent the corresponding standard deviations between the colonies. Shown is a representative experiment out of three independent repeats. No significant difference in growth was observed in cells recovered from the edge and center and recovered in LB (P = 0.6004). Cells harvested directly from the center and edges differed significantly in their growth (P < 0.0005). (**F**) Cells harvested from the edge and the centers were mixed in a 1:1 ratio [mixture]. The cells were grown in a biofilm medium with glutamate as the nitrogen source, and sodium lactate as the carbon source. The mixed samples differed from the other samples by a p-value < 0.001. The results show the average and standard deviation of two independent experiments performed with 10 technical repeats, normalized by spectrophotometer. Equal number of cells taken from the periphery [edge], interior [center] or a 1:1 mixture of both the edge and the center, and grown in a biofilm medium with glutamate as the nitrogen source, and sodium lactate as the carbon source. The mixed samples differed from the other samples by a p-Value < 0.01. (**G**) The cells were grown in a biofilm medium with glutamate as the nitrogen source, and sodium lactate as the carbon source with or without DON (0.1 µM). The mixed samples grown without DON differed from the other samples by a p-Value < 0.05. The edges and mixed samples grown with or without DON differed from each other by a p-value < 0.005. The results are an average of two independent experiments performed with 10 technical repeats. Errors bars represent the corresponding standard deviations.

To examine the metabolic dependence on glutamine of cells located in different regions of a biofilm colony, we compared the growth of cells harvested from the hypoxic center of the biofilm to the growth of cells harvested from the peripheral oxidized dividing edges. We found that bacterial growth in a nitrogen and carbon rich medium was not significantly different between the center and edges populations (Fig. 4A), while when grown on lactate as a sole carbon source, a weak but an apparent growth was observed only in cells taken from the center of the colony (Fig. 4B). On the other hand, when glutamate is provided as the sole carbon and nitrogen source, the growth rate of cells from the edges was significantly higher than the growth of cells from the center (Fig. 4C), supporting differential growth requirement for nitrogen and carbon sources between peripheral and central cells of the biofilm. Consistently, adding DON dramatically restricted the growth of the peripheral cells in a fresh medium while it had no significant effect on the central cells (Fig. 4D). This metabolic memory was lost upon regrowth in a rich medium for 4 hours (Fig. 4E). To further demonstrate that cells residing within the biofilm community can cooperate with each other by sharing nutrients, we grew a mixture of an equal amount of cells harvested from the center and the periphery in a medium containing both glutamate and lactate. As shown in Fig. 4F, a mixture of both cell types grew significantly better than each cell type alone. Interestingly, restricting glutamine levels with DON abolished the growth advantage for the mixture of cells even in the presence of glutamate as a nitrogen source and lactate as a carbon source (Fig. 4G), suggesting that glutamine supplementation provides a more available substrate for growth than glutamine, which has to be synthesized by the biofilm.

**Figure 5:**
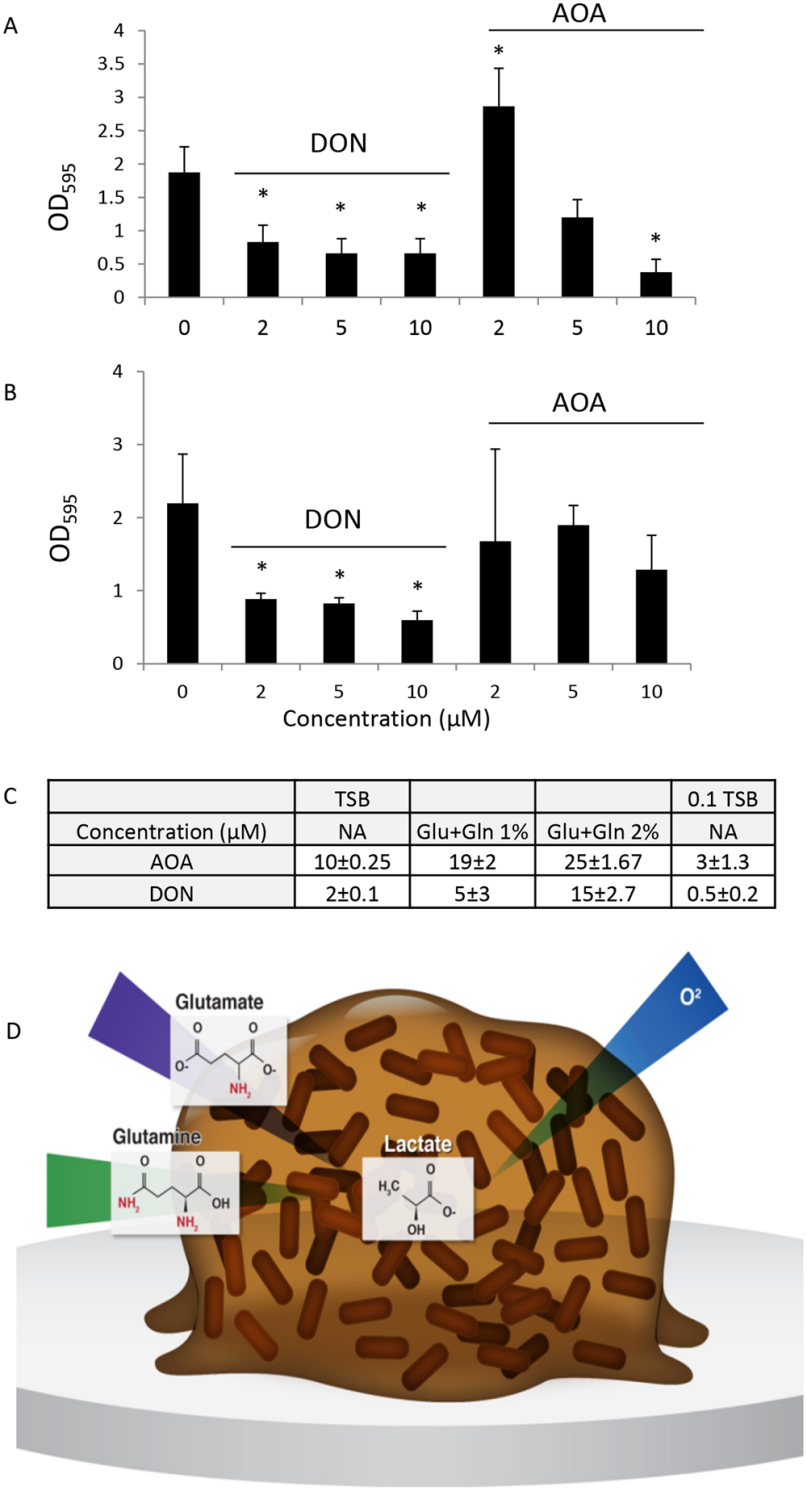
Glutamate and glutamine are essential for biofilm formation. (**A**) Biofilm quantification of *E*. *faecalis* biofilm formation, as judged by crystal violet staining. Biofilms were grown in different concentrations of the inhibitors in TSB-Glucose medium for 24 hrs. *P-value < 0.005 compared to the untreated control. (**B**) Biofilm quantification of *P*.*aeruginosa* biofilm formation, as judged by crystal violet staining. The results are the average for three independent experiments performed with ten technical repeats. Errors bars represent the corresponding standard deviations. Biofilms were grown in different concentrations of the inhibitors in TSB medium for 12 hrs. *P-value < 0.005 compared to the untreated control. (**C**) The change in the minimal biofilm inhibitory concentrations of DON and AHA in different growth media for *E*. *faecalis* biofilm formation. Results are average and standard deviation of one representative experiment out of three, performed with five technical repeats. (**D**) A schematic model representing the gradients of oxygen, glutamine/glutamate and lactate within biofilms, with the center being deficient of oxygen, glutamine and glutamate and enriched with lactate.

Finally, we show that this role of glutamine in biofilm development is not restricted only to B. *subtilis*. Two other species of pathogenic bacteria - Gram-positive *E*. *faecalis* and Gram-negative *P*. *aeruginosa* also depend on glutamine for biofilm formation (Fig. 5A,B) in a glutamine and glutamate dependent manner (Fig. 5C and Table S2), Overall, our results demonstrate that glutamine is an important cue for biofilm development in multiple bacterial species, and hence targeting its’ metabolism might lead to development of novel anti-biofilm therapies.

## Discussion

Bacterial cells assemble into multicellular communities designated biofilms. These multicellular communities may be the primary life-style in which bacteria exist in nature^8^. In the past few decades, biofilms have been extensively characterized owing to their prominent role in disease, due to their increased antibiotic resistance as compared with planktonic cells^14^.

Multiple studies demonstrated that the environmental and physiological conditions are not homogeneous throughout a biofilm^41–43^, and that the metabolic activities of the cells, together with diffusion, result in nutrient concentration gradients and in differential gene expression^43^. Therefore, cells that are growing in biofilms are not only physiologically distinct from planktonic cells, but also become diverse, both spatially and temporally, as biofilm development proceeds. A key issue in restricting biofilm development is identifying the different metabolites whose distribution and synthesis play a cardinal role in biofilm development and differentiation.

We demonstrate here that glutamine and glutamate are essential for the development of biofilms, and that glutamine has a more significant role then glutamate. In continuation to the findings recently described^43^, we show here both a functional requirement and tight expression regulation for glutamine synthesis by the peripheral cells, which are important for biofilm growth, structure and survival (Fig. 5C). Furthermore, our results indicate that biofilm cells preserve a short-term memory of their metabolic activities, effecting their subsequent planktonic growth. This short-term memory of specific metabolic expertise is lost during re-culturing in rich medium, and may be a result of differential accumulation of stable metabolic proteins in the differentiated biofilm cells.

Importantly, from translational perspective, drugs inhibiting glutamine and glutamate synthesis results in disruption of the biofilm structure and biomass, as well as in interference with the metabolic cooperation between the cells residing in different biofilm layers. These results imply that disturbing glutamine and glutamate metabolism could potentially serve to inhibit biofilm formation, an immense need in different ecological, medical and biotechnological settings^71–73^.

## Electronic supplementary material

Supporting Information
