# Supplementary material for: Novel antibiofilm chemotherapies target nitrogen from glutamate and glutamine
Source: Sci Rep. 2018 May 8;8:7097. doi: 10.1038/s41598-018-25401-z (PMC5940852; doi:10.1038/s41598-018-25401-z)
Supplement: Supplementary file 1 — Supporting Information [file 41598_2018_25401_MOESM1_ESM.pdf]

Title: Novel antibiofilm chemotherapies target nitrogen from glutamate and glutamine

Tal Hassanov[1], Iris Karunker[1], Nitai Steinberg[1], Ayelet Erez[2], and Ilana Kolodkin-Gal[1*]

Supporting information

Supplementary Figures and Legends (S1-S4)

Supplementary Tables (S1-S2)

Supplementary References

Figure S1

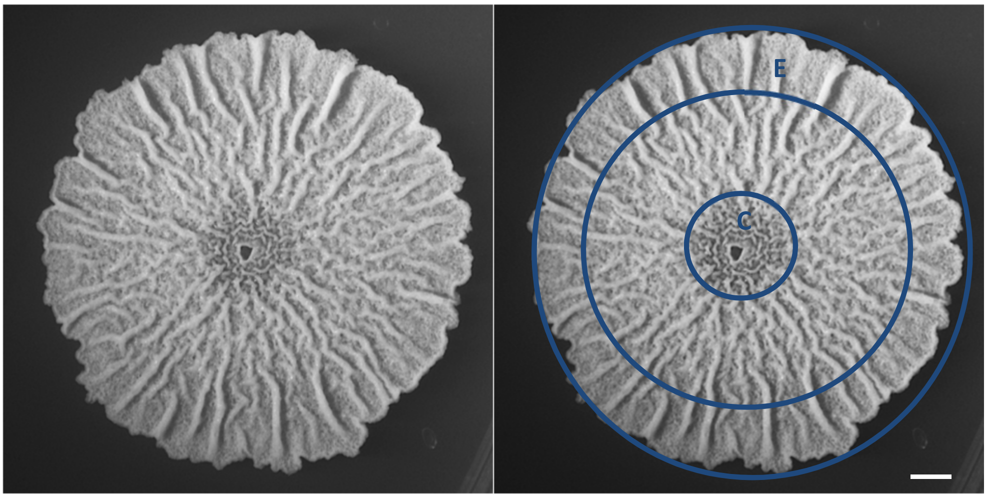

Distinct developmental zones in *B. subtilis* biofilms. Shown are the areas chosen for re-growth analyses. Both the center [C] and the edges [E]. The center indicated as C- is a reproducible thinner circular zone within a colony [1-3]. The edges indicated as E- are reproducible distinct areas which extend up to 0.25 cm from the external boarder of the colony. This zone includes higher wrinkles and ridges which are two-three folds less dense than the interim region[3]. Scale bar corresponds to 2 mm.

Figure S2

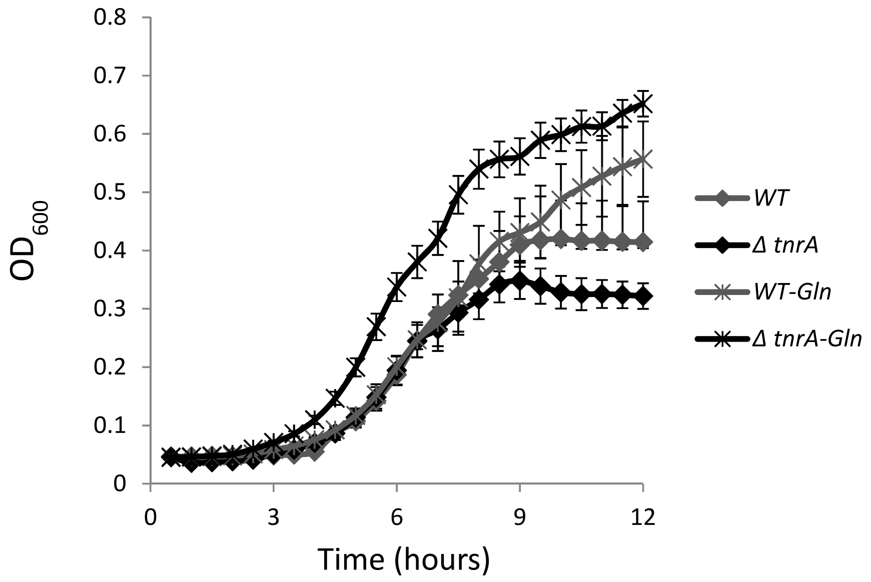

***tnrA* deletion has a subtle effect on planktonic growth.** Shown is the growth of the parental wild-type parental strain and its *tnrA* mutant derivative on a biofilm medium MSgg either not applied or applied with 2% glutamine (Gln). Planktonic growth was assessed as in Figure 1.

Figure S3

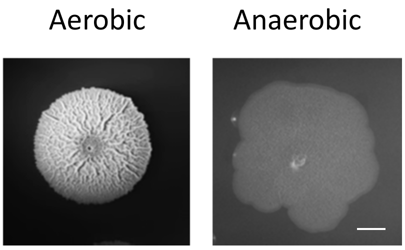

Anaerobic growth prevents complex colony formation Biofilms were grown on solid medium containing MSgg and nitrate source[4] as described in the materials and methods. Anaerobic biofilms were grown in anaerobic chamber. Biofilms were grown for four days at room temperature. Images of colonies and pellicles were acquired with a stereomicroscope (Zeiss) and were optimized for contrast and brightness, with adjustments kept consistent between control and treatments. Scale bar corresponds to 2 mm

Figure S4

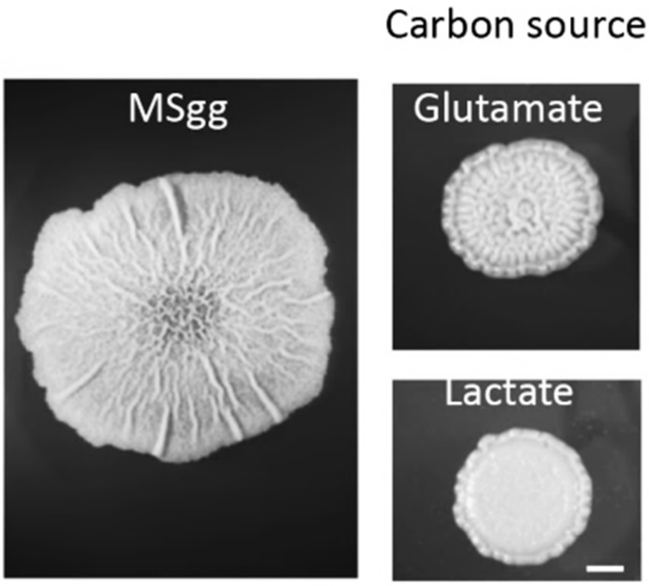

The utilization of glutamine and lactate and its impact on biofilm morphology. Biofilms were grown on either the solid biofilm MSgg medium, MS-Glutamate in which only glutamate served as a carbon and a nitrogen source, or on MS-lactate, where sodium lactate was applied as a carbon source (0.5%) and ammonium chloride served as a nitrogen source. Scale bar represents 2 mm

Table S1

Concentration (μM)			Gln 0.5%		Gln 1%		Gln 2%	
	MBIC	MIC	MBIC	MIC	MBIC	MIC	MBIC	MIC
DON	0.1±0.07	2±0.5	0.3±0.09	1.9±0.6	0.6±0.05	3±0.07	0.8±0.09	3.2±0.05

Biofilms of *B. subtilis* were grown on liquid biofilm MSgg medium either with shaking or without in the presence of increasing glutamine concentrations in triplicates. Floating biofilm inhibition (MBIC) and growth inhibitory concentration (MIC) were determined. MBIC was determined as a minimal inhibitor concentration that induced a 12 hrs inhibition in pellicle formation. MIC was determined as a minimal inhibitor concentration that induced an increase of at least six hours in the lag time. Results are an average and standard deviation of three independent repeats.

Table S2

	TSB			0.1 TSB
Concentration (μM)	NA	Glu+Gln 1%	Glu+Gln 2%	NA
AOA	15±3	18±6	30±4	6±2.7
DON	2±0.3	8±2.3	12±1.6	1±0.3

Shown is the change in the minimal concentration of biofilm inhibitors between different growth media for *P.aeruginosa* biofilm formation, as judged by crystal violet staining. The results are Results are average and standard deviation of one representative experiment out of three, performed with five technical repeats. Biofilms were grown in different concentrations of the inhibitors in either TSB or 10% TSB medium for 12 hrs. * P-value <0.01 compared to the untreated control. The inhibitor group in the presence addition of glutamate and glutamine (Glu+Gln) differed significantly from each inhibitor alone (P-value<0.005)

Supporting references

1 Asally, M. *et al.* Localized cell death focuses mechanical forces during 3D patterning in a biofilm. *Proceedings of the National Academy of Sciences of the United States of America* **109**, 18891-18896, doi:10.1073/pnas.1212429109 (2012).

2 Vlamakis, H., Aguilar, C., Losick, R. & Kolter, R. Control of cell fate by the formation of an architecturally complex bacterial community. *Genes & development* **22**, 945-953, doi:10.1101/gad.1645008 (2008).

3 Bloom-Ackermann, Z. *et al.* Toxin-Antitoxin systems eliminate defective cells and preserve symmetry in Bacillus subtilis biofilms. *Environmental microbiology* **18**, 5032-5047, doi:10.1111/1462-2920.13471 (2016).

4 Nakano, M. M. & Zuber, P. Anaerobic growth of a "strict aerobe" (Bacillus subtilis). *Annual review of microbiology* **52**, 165-190, doi:10.1146/annurev.micro.52.1.165 (1998).
